# Cerebellar mutism syndrome in children with brain tumours of the posterior fossa

**DOI:** 10.1186/s12885-017-3416-0

**Published:** 2017-06-21

**Authors:** Morten Wibroe, Johan Cappelen, Charlotte Castor, Niels Clausen, Pernilla Grillner, Thora Gudrunardottir, Ramneek Gupta, Bengt Gustavsson, Mats Heyman, Stefan Holm, Atte Karppinen, Camilla Klausen, Tuula Lönnqvist, René Mathiasen, Pelle Nilsson, Karsten Nysom, Karin Persson, Olof Rask, Kjeld Schmiegelow, Astrid Sehested, Harald Thomassen, Ingrid Tonning-Olsson, Barbara Zetterqvist, Marianne Juhler

**Affiliations:** 1 Department of Neurosurgery, Rigshospitalet, Copenhagen, Denmark; 2 Department of Pediatrics and Adolescent Medicine, Rigshospitalet, Copenhagen, Denmark; 3 Department of Neurosurgery, St. Olavs Hospital, Trondheim, Norway; 4 Department of Paediatrics Lund Skåne University Hospital, Lund, Sweden; 5 Department of Pediatrics, Aarhus University Hospital, Skejby, Aarhus, Denmark; 6 Department of Women’s and Children’s Health, Karolinska Universitetssjukhuset, Stockholm, Sweden; 7 Posterior Fossa Society, https://www.posteriorfossa.org; 8 Department of Oncology and Palliation, North Zealand Hospital, Hillerød, Denmark; 9 Center for Biological Sequence Analysis, Technical University of Denmark, Kgs. Lyngby, Denmark; 10 Department of Neurosurgery, Karolinska University Hospital, Stockholm, Sweden; 11 Department of Neurosurgery, Helsinki University Hospital, Helsinki, Finland; 12 Department of Neuroradiology, University Hospital of Copenhagen, Rigshospitalet, Copenhagen, Denmark; 13 Department of Child Neurology, Helsinki University Central Hospital, Helsinki, Finland; 14 Department of Neuroscience, Neurosurgery, Akademiska sjukhuset, Uppsala, Sweden; 15 Child and Youth Rehabilitation Centre, Habilitation and Technical Aid, Lund, Sweden; 16 Institute of Clinical Medicine, University of Copenhagen, Copenhagen, Denmark; 17 Division of Pediatric Hematology/Oncology, Perlmutter Cancer Center, Univesity Langone Medical Center, New York, USA; 18 Department of Pediatrics, St. Olavs Hospital, Trondheim, Norway; 19 Department of Clinical Intervention and Technique, Karolinska Institute, Stockholm, Sweden

**Keywords:** Cancer, Paediatric, Children, Cerebellar mutism, CMS, Brain tumour, Cerebellum, Posterior fossa syndrome, Genetics, Neurosurgery

## Abstract

**Background:**

Central nervous system tumours constitute 25% of all childhood cancers; more than half are located in the posterior fossa and surgery is usually part of therapy. One of the most disabling late effects of posterior fossa tumour surgery is the cerebellar mutism syndrome (CMS) which has been reported in up to 39% of the patients but the exact incidence is uncertain since milder cases may be unrecognized. Recovery is usually incomplete. Reported risk factors are tumour type, midline location and brainstem involvement, but the exact aetiology, surgical and other risk factors, the clinical course and strategies for prevention and treatment are yet to be determined.

**Methods:**

This observational, prospective, multicentre study will include 500 children with posterior fossa tumours. It opened late 2014 with participation from 20 Nordic and Baltic centres. From 2016, five British centres and four Dutch centres will join with a total annual accrual of 130 patients. Three other major European centres are invited to join from 2016/17. Follow-up will run for 12 months after inclusion of the last patient. All patients are treated according to local practice. Clinical data are collected through standardized online registration at pre-determined time points pre- and postoperatively. Neurological status and speech functions are examined pre-operatively and postoperatively at 1–4 weeks, 2 and 12 months. Pre- and postoperative speech samples are recorded and analysed. Imaging will be reviewed centrally. Pathology is classified according to the 2007 WHO system. Germline DNA will be collected from all patients for associations between CMS characteristics and host genome variants including pathway profiles.

**Discussion:**

Through prospective and detailed collection of information on 1) differences in incidence and clinical course of CMS for different patient and tumour characteristics, 2) standardized surgical data and their association with CMS, 3) diversities and results of other therapeutic interventions, and 4) the role of host genome variants, we aim to achieve a better understanding of risk factors for and the clinical course of CMS - with the ultimate goal of defining strategies for prevention and treatment of this severely disabling condition.

**Trial registration:**

Clinicaltrials.gov: NCT02300766, date of registration: November 21, 2014.

## Background

### Incidence and definition of CMS

Central nervous system (CNS) tumours account for 25% of all cancers in children and over half of these are located in the posterior fossa [1]. For most of these patients, treatment includes surgery. Posterior fossa tumours in children are associated with high risk of chronic neurological and neurocognitive disability [2–6]. The cerebellar mutism syndrome (CMS) refers to the constellation of transient mutism, ataxia, hypotonia and irritability following surgery for cerebellar or fourth ventricle tumours in children and adolescents [7]. Although some patients may recover completely, recovery may be prolonged, and many are left with permanent disabling sequelae in the form of e.g. dysarthria, dysfluency, slowed speech rate and ataxia. Many may in addition be burdened by emotional problems and lower IQ [8–12].

The spectrum of CMS definitions varies greatly [7, 12–14], leading to differences in reported incidence and uncertainties about recovery. Incidence figures thus range from 8% [15, 16] to 32% [17] in children with any kind of cerebellar tumour when a variety of definitions are used, compared to 24% [12] to 39% [18] in patients with medulloblastomas using a more precise CMS definition. A recent study of 148 children with cerebellar tumours found that the overall incidence of the broader Posterior Fossa Syndrome was 28%, subdivided by tumour pathology into 40% for medulloblastoma, 16% for astrocytoma and 20% for ependymoma [19]. The CMS definition created by the Neurology Committee of the Children’s Cancer Group in USA in 1993 is currently the only one associated with a specific scoring scale [12] and is used in this project.

### Risk factors and prevention

Cerebellar mutism is thought to be caused by bilateral disturbance of the dentate nuclei and/or their efferents [16, 20–24]. Ataxia and irritability together with other cognitive, affective and motor symptoms that are frequently observed in CMS patients are caused by damage to various parts of the cerebellum and cerebello-cerebral pathways passing through the brainstem [25–28]. This can result in secondary diaschisis of supratentorial brain areas due to lack of excitatory input from cerebellum [29–31].

*Known risk factors* are brainstem involvement by the tumour, midline location and tumour type; thus the incidence in children with medulloblastoma is two to three times higher than for astrocytoma or ependymoma but the biological mechanisms behind these associations are uncertain [12, 19, 22, 32–35]. Recently *proposed risk factors* include brainstem compression by the tumour, pre-operative language impairment, low socioeconomic level of the families and left-handedness [24, 36–38]; however, these remain to be verified on large patient cohorts. Tumour size, neurosurgical techniques and approaches, radical resection and younger age at diagnosis are *uncertain risk factors*, as previous studies have been inconclusive [12, 23, 32–35, 39, 40]. Gender, hydrocephalus, post-operative central nervous system infections, type of neurosurgeon (adult/paediatric), and oedema/swelling of the cerebellum have not been significantly correlated to CMS and are considered *unlikely risk factors* [12, 16, 34, 35, 41, 42].

In traumatic brain injury common host genomic variants are related to the severity of symptoms and degree of recovery [43–45]. Similar associations are likely for surgical brain injury, but such studies have not been performed for CMS.

### Non-surgical treatment

Supportive speech and rehabilitation therapy is often offered to patients with CMS, but the benefit hereof has not been demonstrated. No publications exist on systematic approaches to pharmacological neuroprotection, and pharmacological interventions are only sporadically reported in the literature [46–50]. Glucocorticosteroids are routinely given to most patients pre-, intra- and post-operatively to reduce inflammation and edema [51] [52], but there is no consensus recommendation and the impact on the clinical course of CMS is undetermined.

### Aims

The study focuses on the risk factors for development and severity of CMS including surgery (approaches, techniques and tissue and vascular damage, re-operation) and host genome variants. The aims of this study are thus to describe differences in incidence, severity and clinical course of CMS related to:

1. Clinical factors: gender, age, handedness, speech, language and neuropsychological abilities before and after surgery.
2. Tumour factors: histological tumour type and tumour location
3. Surgical factors: Surgical strategy and surgical trauma including access routes, removal technique, tissue and vascular injury, bleeding and primary surgery vs. re-operation
4. Non-surgical interventions: glucocorticosteroids, other symptomatic medication and chemo- and radiotherapy
5. Host genome variants

## Methods/design

### Design

This open observational study is registered at Clinicaltrials.gov (file NCT02300766) and EANS (http://www.sbns.org.uk/index.php/research/eu-multi-centre-trials). All children younger than 18 years with a tumour in the posterior fossa requiring surgery or open biopsy at one of the participating centres will be included following informed consent. Patients who have received surgery, chemotherapy and/or radiotherapy previously are also eligible. The study will run for five years with a targeted sample size of 500 patients. It opened late 2014 with participation from 20 Nordic and Baltic centres. From 2016, five British centres and four Dutch centres will join leading to an expected annual accrual of 130 patients. Three other major European centres are invited to join from 2016/17. The target of 500 patients is expected to be reached in 2018. Patients will be followed for 12 months after inclusion of the last patient, and the study will thus be completed during 2019.The participating centres provide surgery and supportive care according to local practice and register all study information in an online database developed specifically for this study. Consensus concerning the study aims, study design and data registration was achieved at three international planning meetings among the initiating centres during 2013. The annual enrolment from each country will be compared to the number of registered patients in the national cancer registries to document the inclusion rate and representativeness.

### Primary endpoint

The primary endpoint is incidence and severity of CMS. Symptoms and severity are scored according to the CMS survey published by Robertsons et al. [12]. Our main focus is the impact of different surgical tumour approaches. We hypothesize that 1) minimally traumatic techniques and 2) sparing the dentate nuclei and their efferents will be associated with a 50% reduced risk of CMS when compared to more invasive tumour removal approaches. Furthermore, we hypothesize that the risk of developing CMS is higher after re-operation(s) compared to primary surgery.

### Secondary endpoint

The secondary endpoint is incidence of “reduced speech output” defined as “severely reduced speech production limited to single words or short sentences which can only be elicited after vigorous stimulation” [19]. The risk of reduced speech output will be related to different surgical approaches with the underlying hypothesis that damage to the dentate nuclei and/or their efferents increases the risk.

Furthermore, we want to explore the following:

#### Genetics

We will analyse the role of host genome variants on development, severity and recovery from CMS by carrying out broad genetic pathway profiling of all study participants using both non-CMS cases from the study cohort and non-CNS tumour patients as controls. Genotyping will use single nucleotide polymorphism (SNP) exome enriched arrays (e.g. Illumina Omni2.5-exome platform). We will apply agnostic genome-wide association studies (GWAS) as well as more complex pathway analyses. Thus, we will interrogate combined effects of multiple SNPs acting in the same pathways or protein-protein interaction complexes using our validated non-linear machine learning algorithm (artificial neural networks approach) [53, 54], which allows testing of a large range of pathways from various databases. This approach will yield hypotheses easier to test across cohorts and also provide mechanistic insights. We hypothesize that host genome variants explain at least 50% of the variation in incidence of CMS and at least 40% of the variation in severity, duration and level of recovery from the CMS.

#### Other non-surgical treatments including glucocorticosteroids

The possible effects of chemo- and radiotherapy on recovery from CMS will be investigated. We hypothesize that chemo- and radiotherapy delay recovery from CMS. For descriptive documentation purposes we also ask for information on medications given specifically to treat the symptoms of CMS.

We hypothesize that glucocorticosteroids 1) given preoperatively protect against CMS due to reduced oedema; 2) given intraoperatively increase the risk of CMS due to worsening of acute neurological injury by hyperglycaemia; 3) given postoperatively negatively affect the course of CMS as earlier studies have shown a negative effect of glucocorticosteroids on the outcome of traumatic brain injury [55, 56]. It may be expected that most patients receive glucocorticosteroids at all 3 time points which would make it difficult to assess added positive and negative effect in the same patients.

#### Tumour type

The incidence of the CMS will be correlated to tumour histology using the 2007 WHO classification. We hypothesize that the risk of CMS is highest among patients with medulloblastoma. With increasing focus on subtyping of medulloblastoma [57] this additional classification may later be added to the risk factor analysis.

#### Neuroradiology

Tumour location, enhancement pattern, invasiveness and growth velocity may affect the risk and severity of CMS [12, 18, 58]. Accordingly, we hypothesize that a statistical risk of CMS may be predicted by defining specific neuroradiological features [59–61]. Likewise, postoperative neuroradiological features could give prognostic information about probable degree of recovery.

#### Handedness

We will determine whether the risk of the CMS varies according to handedness. We hypothesize that the risk of CMS is increased in left-handed patients, and possibly even more so in patients with medulloblastoma [24].

#### Language and speech

Our hypothesis that preoperative speech and language impairment increases the risk of postoperative speech and language deficits will be explored by recording pre- and postoperative speech (e.g. articulation, prosodic features and voice) and language (e.g. word finding difficulties, fluency and narrative ability) statuses and relating these to incidence and course of CMS. All speech recordings will be analysed nationally by speech therapists.

### Registration of data

The following data will be registered at five time points by online standard registration forms:

#### 1. Preoperatively

Hospital, country, patient related variables such as date of birth, handedness, comorbidities, bilingualism, gender and date of diagnosis, medical history and preoperative neurological status. A speech and language test will be performed and recorded. If the patient is younger than two years a bedside assessment of speech will be performed instead of a formalized test. A two millilitre blood sample for genetic analysis will be collected.

#### 2. Postoperatively within 72 h of surgery

Surgery related variables such as date, patient position during surgery, surgical approach, tumour removal method (én bloc, piecemeal or ultrasonic aspiration), duration and course of operation, damage to non-tumour tissue, complications, technology employed (endoscopy, neuronavigation, electrophysiological monitoring etc.), surgeon’s estimate of tumour resection extent and presence of preoperative hydrocephalus.

#### 3. Postoperatively within one to four weeks from surgery

Approximately one to two weeks post-operatively: neurological examination, postoperative speech and language status including speech and language recording or bedside assessment and medications used for treatment of CMS.

Approximately four weeks post-operatively: Development and treatment of postoperative intracranial haematoma and hydrocephalus, leakage of cerebrospinal fluid and need for ventilator. These complications are usually seen earlier but we wait until the fourth post-operative week to register these in order to ensure no complications are missed.

#### 4. Postoperatively two months after surgery

Neurological examination, CMS-survey, speech and language recording or bedside assessment and any medications given to treat CMS since last registration.

#### 5. Postoperatively twelve months after surgery

Neurological examination, speech and language status including speech and language recording or bedside assessment, medications given since last registration to treat CMS, chemo- and/or radiotherapy, neuropsychological assessment(s) if performed, final neuropathological classification of tumour, and additional neuroimaging performed since the first follow-up. Copies of the neuroimaging and descriptions performed pre- and postoperatively will be collected for central review.

### Acute and repeated neurosurgery

In case of emergency surgery (e.g. due to risk of incarceration or coma) information about the study and invitation to participate can be given within seven days postoperatively. These patients will be included in all parts of the study except for the recording and analysis of preoperative speech and language status.

**Fig. 1:**
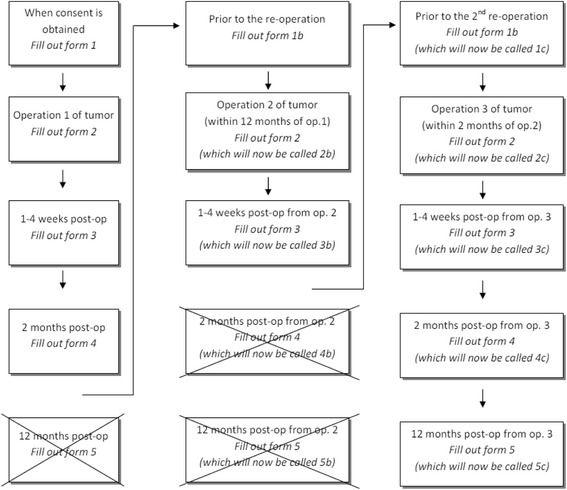
The follow up process in case of repeated surgeries

In cases with repeat tumour surgery during the twelve months follow-up, the patient can re-enter the study and start a new follow-up programme (Fig. 1, Repeated Surgeries). A new pre-operative registration is then performed corresponding to the re-operation. Post-operative registrations will be performed again, and used in the analysis of risk related to first versus further surgeries. If surgery is performed again after the twelve months follow-up period, the patient will be re-invited to participate in the study.

### Statistical considerations

In accordance with our surgical hypothesis of 50% risk reduction by less traumatic techniques, and assuming that 35% of the patients are operated using an approach with a low risk of CMS (assumed to be 10%) and the remaining 65% of patients are operated using other approaches (assumed carrying a 20% risk), a total of 450 patients have to be included to identify a 5% significance level and 80% power. Based on a projected overall risk of CMS of 20%, an estimated frequency of a specific SNP of 30%, and a projected doubled risk of CMS with this particular SNP, we will need to include a total of 343 patients to identify such a genetic predisposition at a 5% significance level with 90% power.

## Discussion

The study will be the largest prospective international study on CMS to date, and the first one to 1) systematically register surgery, use of steroids, standardized speech samples and 2) to investigate the influence of host genome. Detailed information on neuroradiological features, tumour and patient characteristics (incl. Handedness and pre-language impairment) will also be gathered, and may help further elucidate the incidence and clinical course of the syndrome for various patient and tumour types.

On-line registration compliance rates to Nordic/Baltic multicentre trials are in general above 95% [62]. Furthermore, we will implement an automated email reminder system at the four follow-up time points and the project coordinator and data manager will validate all data inputs, request clarifications and updates for unclear or missing data, and secure that DNA of sufficient quality is received, processed and stored for later host genome analyses.

Currently, a randomized intervention study is unrealistic due to limited data supporting any specific neurosurgical approach and given the diversity of tumour subtypes, localisation and invasiveness. However, such a randomisation may be realistic if the present study does not clearly identify surgical approaches with statistically significant reduced risks of CMS.

## Abbreviations

CMS: Cerebellar mutism syndrome
CNS: Central nervous system
GWAS: Genome-wide association studies (GWAS)
SNP: Single nucleotide polymorphism
